# Polydopamine-Coated Magnetic Iron Oxide Nanoparticles: From Design to Applications

**DOI:** 10.3390/nano12071145

**Published:** 2022-03-30

**Authors:** Giulia Siciliano, Anna Grazia Monteduro, Antonio Turco, Elisabetta Primiceri, Silvia Rizzato, Nicoletta Depalo, Maria Lucia Curri, Giuseppe Maruccio

**Affiliations:** 1 Omnics Research Group, Department of Mathematics and Physics “Ennio De Giorgi”, University of Salento, Via per Monteroni, 73100 Lecce, Italy; annagrazia.monteduro@unisalento.it (A.G.M.); silvia.rizzato@unisalento.it (S.R.); 2 Omnics Research Group, Institute of Nanotechnology, CNR-Nanotec, Via per Monteroni, 73100 Lecce, Italy; antonio.turco@nanotec.cnr.it (A.T.); elisabetta.primiceri@nanotec.cnr.it (E.P.); 3 Institute for Chemical and Physical Processes, CNR-IPCF SS Bari, Via Orabona 4, 70126 Bari, Italy; n.depalo@ba.ipcf.cnr.it (N.D.); lucia.curri@ba.ipcf.cnr.it (M.L.C.); 4 Department of Chemistry, University of Bari, Via Orabona 4, 70126 Bari, Italy

**Keywords:** magnetic nanoparticles, iron oxide nanoparticles, polydopamine, surface functionalization, bioinspired nanomaterials

## Abstract

Magnetic iron oxide nanoparticles have been extensively investigated due to their applications in various fields such as biomedicine, sensing, and environmental remediation. However, they need to be coated with a suitable material in order to make them biocompatible and to add new functionalities on their surface. This review is intended to give a comprehensive overview of recent advantages and applications of iron oxide nanoparticles coated by polydopamine film. The synthesis method of magnetic nanoparticles, their functionalization with bioinspired materials and (in particular) with polydopamine are discussed. Finally, some interesting applications of polydopamine-coated magnetic iron oxide nanoparticles will be pointed out.

## 1. Introduction

In the last decades, magnetic nanoparticles (MNPs) have been extensively investigated due to their various applications in fields such as biomedicine [1], hyperthermia [2], catalysis [3], wastewater treatment [4], and spintronics [5,6]. Among them, iron oxide nanoparticles have attracted major attention because of their magnetic properties, chemical stability, tuneable morphology, and ease of surface functionalization [7]. However, these nanoparticles need to be coated with a suitable material in order to prevent agglomeration or to add new functionalities on their surface. Surface modification can be carried out in different ways and using various biomaterials [8]. Typical purposes are to obtain in a single step reaction: the available material, the use of water as a solvent, and a coating exploitable for secondary functionalization with specific molecules. Taking inspiration from the adhesion properties of mussels, a uniform coating platform based on the use of dopamine has been developed, leading to the use of polydopamine as a novel coating material [9]. Polydopamine is a highly biocompatible bioinspired material that can be easily deposited on various substrates with a good control on film thickness [10]. The functional groups on its surface (catechol, carboxylic groups amine and imine) can be used to bind specific molecules or to load transition metal ions. These unique properties make polydopamine convenient not only as a coating material, but also as an innovative biomaterial with applications in the fields of chemistry, biology, and material science [11].

The aim of this review is to provide a global overview of the properties, advantages, and applications of iron oxide nanoparticles coated by polydopamine films. First of all, we will introduce the synthesis procedure of magnetic nanoparticles, their functionalization with biomaterials, and discuss the synthesis approaches of polydopamine, the factors that influence its polymerization, and its chemical properties. Then, applications of polydopamine-coated magnetic iron oxide nanoparticles will be discussed.

## 2. Magnetic Nanoparticles: Synthesis and Functionalization

Magnetic nanoparticles with specific features can be obtained using ferromagnetic elements such as Fe, Ni, Co or metal oxides (Fe_2_O_3_, Fe_3_O_4_), alloys (CoPt, FePt), and ferrites (MnFe_2_O_4_, CoFe_2_O_4_).

The electronic and magnetic properties of nanoparticles depend on their size [12]. In particular, finite-size effects derived from the electron quantum confinement, and surface effects related to the symmetry of the crystal structure, dominate the nanoparticles’ magnetic properties. Large particles have a multi-magnetic domain structure, with a remanent magnetization in the absence of an external magnetic field, and exhibit a ferromagnetic behavior. Decreasing the particle size to the nanoscale (typically around 10–25 nm) results in a single magnetic domain structure with all the spins lined in the same direction, but with a superparamagnetic behavior [13]. When an external magnetic field is absent, superparamagnetic nanoparticles exhibit zero magnetization, no coercivity, and less tendency to agglomerate at room temperature, which makes them good candidates for biomedical and adsorptive applications. However, for magnetic separation, particles with ferromagnetic properties are mainly used [14].

The strong connection between the size, the shape, and the magnetic properties of the nanoparticles leads to the development of a wide number of synthetic procedures to achieve high crystallinity, a narrow size distribution, uniform morphology, and tuneable properties [15]. Magnetic nanoparticles can be obtained by physical or chemical methods. Physical strategies include top-down approaches, leading to nano-sized materials from bulk materials (molecular beam epitaxy [16], chemical vapour deposition [17], and spray pyrolysis [18]). The main disadvantage of these techniques is the formation of powders with a wide side distribution. Chemical methods involve, on the contrary, bottom-up approaches, since they use molecular precursors to synthetize nanocrystals. Chemical methods for the synthesis of high-quality magnetic nanoparticles include co-precipitation [19], microemulsion [20], hydrothermal treatment [21], and thermal decomposition in the presence of molecular precursors [22]. The co-precipitation method is extensively used for the synthesis of MNPs, with a good control on size and magnetic properties for biomedical applications. However, MNPs obtained by this technique tend to agglomerate because of their small particle size [19]. The microemulsion technique allows for the obtainment of MNPs, with a good control over size and composition and high saturation magnetization. However, the type of surfactant used affects nanoparticles’ properties, and represents a great disadvantage [20]. The hydrothermal or solvothermal method is mainly used for the synthesis of ultrafine powder and crystals of different materials. For example, Zheng et al. [23] have reported the hydrothermal synthesis of Fe_3_O_4_ NPs in the presence of sodium bis (2-ethylhexyl) sulfosuccinate as surfactant. The main disadvantage associated with this synthetic route is that nanoparticles smaller than 10 nm in size cannot be obtained [21]. Among the various chemical methods used for the fabrication of MNPs, the thermal decomposition of organometallic precursors in the presence of stabilizing agents such as surfactants best allows the synthesis of inorganic nanoparticles in a wide range of composition, including oxides, metals, and semiconductors, with a good control of their size, shape, size dispersion, crystallinity, and, accordingly, the resulting physicochemical properties. MNPs obtained by means of such synthetic approaches are dispersible in organic solvent, and require post-synthetic treatments for their application in biological fields [22]. Alivisatos et al. [24] reported the synthesis of maghemite nanocrystals with size of 4–10 nm by the thermal decomposition of iron cupferron complexes (FeCup_3_). Very recently, the γ-irradiation method, commonly named as the radiolytic method, has emerged as a new green synthetic route for magnetic oxides, exploiting the interaction between high energy γ-photons and an aqueous phase [25]. Recently, Jurkin et al. [26] have reported for the first time the synthesis of δ-FeOOH nanodiscs by γ-irradiation of a deoxygenate iron(III) chloride alkaline solution in the presence of diethylaminoethyl-dextran (DEAE-dextran) polymer, which is able to disperse the NPs and form colloidal solutions rather than suspensions.

Magnetic nanoparticles derived from iron, in the form of magnetite (Fe_3_O_4_) or maghemite (γ-Fe_2_O_3_), are the most extensively studied in the last decades, and have become promising candidates for various applications because of their magnetic properties, chemical stability, low toxicity, biological compatibility, tuneable size, and particle shapes that can be controlled by varying the synthesis conditions, as well as the fact that they can be easily coated by surface functionalization [27].

**Figure 1:**
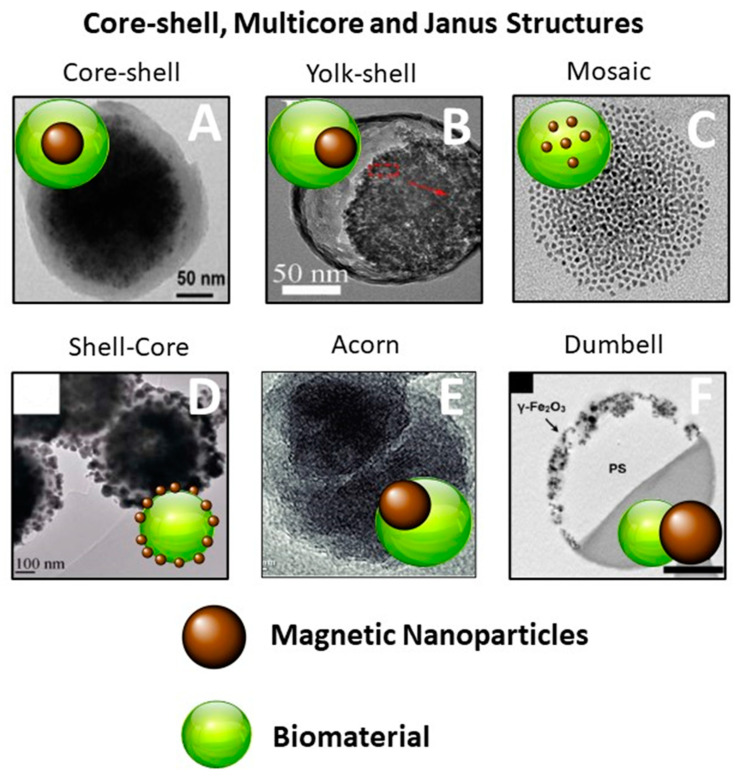
Distinct structures of magnetic bionanocomposites: (**A**) core-shell Fe_3_O_4_ polydopamine nanoparticles (Fe_3_O_4_@PDA NPs) (reproduced with permission from ref. [45]. Copyright 2017 Elsevier); (**B**) Polydopamine@upconversion nanoparticle@mesoporous silica yolk-shell nanoparticles (PDA@UCNP@mSiO_2_ NPs) (reproduced with permission from ref. [46]. Copyright 2020 Elsevier); (**C**) Mosaic Fe_3_O_4_ polydopamine nanoparticles; (**D**) Au speckled SPION@SiO_2_ NPs (reproduced with permission from ref. [33]. Copyright 2020 John Wiley and Sons); (**E**) Polyethylene glycol (PEG) stabilized MnFe_2_O_4_@MnO Janus nanoparticles (reproduced with permission from ref. [48]. Copyright 2003 Royal Society of Chemistry); (**F**) Polymer-stabilized ferromagnetic γ-Fe_2_O_3_ dumbell nanoparticles (reproduced with permission from ref. [49]. Copyright 2013 ACS Publications).

For most applications, the chemical stability of magnetic nanoparticles is crucial in order to prevent agglomeration, precipitation, or oxidation. Moreover, their surface functionalization is essential not only to make them stable against degradation, but also to convey additional properties that enable their specific activity towards target cells, such as tumor cells in order to address hyperthermia, towards biological ligands for the development of electrochemical sensors, and also towards pollutants for the uptake of contaminants from water, thus leading to the fabrication of various nanocomposites with applications in many technological fields [28,29,30,31,32]. The strategies developed for the protection of magnetic nanoparticles, their surface engineering, and their integration in functional structures and materials, can be divided into two major groups: surface coating with inorganic materials (silica shell [33,34], carbon [35,36], metals [37]) and coating with organic materials (surfactants, polymers [38,39,40,41]). In recent years, biopolymers including cellulose, alginate, chitosan, polyethylene glycol (PEG), and synthetic eumelanin-type biopolymers such as mussel-inspired polydopamine (PDA) have received much more attention for MNPs coating owing to their physicochemical properties, which are useful for different applications in various research areas. Surface modification of MNPs can be carried out by two main strategies, i.e., in situ and ex situ processes [42]. In the case of in situ surface functionalization, the coating is carried out during the synthesis of nanoparticles and it starts at the same time of nucleation, avoiding further growth of MNPs [42]. Generally, nanocomposites obtained this way have core-shell or mosaic structures [43] with a variable polymer shell in terms of morphology and thickness. The ex situ surface functionalization procedure, on the contrary, relies on two different stages: the synthesis of nanoparticles and their successive coating with biopolymers, allowing for better control of the nanocomposites’ morphology. In both cases, the interactions attending the adsorption mechanism of biopolymers on the surface of MNPs are mainly electrostatic and hydrophobic interactions, and hydrogen bonding [42]. All strategies available for the surface functionalization of MNPs lead to several magnetic bio-nanocomposites characterized by different structures, including core-shell, shell-core-shell, multicores or matrix-dispersed structures, and Janus-type hetero-structures [44,45,46,47,48,49] (Figure 1).

## 3. Polydopamine Functionalized Iron Oxide Nanoparticles: Synthesis and Structures

Among the various biomaterials used for the protection and functionalization of magnetic nanoparticles, polydopamine—a mimic of the adhesive foot protein secreted from mussels—has stimulated extensive research in recent years for the surface modification of many inorganic and organic materials, because it shows much more flexibility and designability in the target structures compared to other biopolymers, and has singular features and physicochemical properties. Since polydopamine is the major pigment of eumelanin, it is biocompatible and displays many characteristics of natural melanin in terms of optical (UV absorption as photoprotective agent) and electrical properties [50]. One of the most exploited properties of polydopamine is its strong adhesion to all types of substrates, thanks to the many functional groups, such as imine, amine, and catechol incorporated in its structure. The catechol moieties in PDA have a certain redox activity that can be used both for transition metal binding and for covalent bonding with specific molecules, leading to the fabrication of diverse hybrid materials with powerful reducing capability towards metal ions such as Mn^2+^, Cu^2+^, and Zn^2+^. Moreover, the functional groups present in its chemical structure can react with various molecules, allowing the manufacture of heterostructures with applications in different fields. Finally, PDA has excellent biocompatibility, a crucial factor for specific applications in the biomedical field [50].

In light of these properties, a wide number of polydopamine-derived hybrid materials have been developed for diverse applications, including energy (solar cells [51], catalysts [52], supercapacitors [53]), the biomedical field (cells adhesion [54], antibacterial activity [55], photothermal therapy [56], bioimaging [57], drug delivery [58]), water treatment [59,60], and sensing [61,62,63].

The synthesis of polydopamine/iron oxides (PDA/Fe_3_O_4_) core-shell nanoparticles has been widely investigated in recent years. PDA/Fe_3_O_4_ core-shell nanospheres can be synthetized by the precipitation method [59], with PDA nanoparticles acting as templates. On the other hand, introducing a dopamine solution into an Fe_3_O_4_ suspension can result in magnetic nanoparticles coated by a polydopamine shell with a good control on the shell thickness. In these conditions, dopamine could form –COO–NH_2_–ion pairs because of the carboxyl groups on Fe_3_O_4_ surface, and could generate a polymeric shell under basic conditions, leading to the formation of well-defined core/shell structures [64]. There are advantages and limitations related to these two approaches. In particular, the first method is useful for synthesizing iron oxide nanostructures after removing polydopamine. On the other hand, the second approach presents three valuable features. First, multicore nanostructures can be obtained, where polydopamine shell encapsulates magnetic nanoparticles. Second, since magnetic iron oxide nanoparticles tend to aggregate and can biodegrade when they are in biological systems, polydopamine shell can prevent their biodegradation and their direct contact with biological systems. Moreover, polydopamine shell, thanks to its chemistry and reducing ability, is a versatile platform for the surface modification of the inorganic core.

The polymerization process of PDA shell around the surface of iron oxide nanoparticles is affected by parameters such as the dopamine monomer concentration, the pH value, and the type of buffer and oxidation agent used. Usually, polydopamine is synthetized by a solution oxidation method, whereby the dopamine monomers (typically dopamine hydrochloride), added into an alkaline solution (generally tris(hydroxymethil)-aminomethane (Tris) buffer (pH 8.5)), are oxidized and spontaneously self-polymerize. The polymerization process can be easily monitored by a color change of the solution, from colorless to deep brown. Dopamine concentration considerably affects the morphology of PDA nanoparticles and the characteristics of film deposition. Increasing the dopamine monomer concentration from 0.1 to 5 g L^−1^ results in an increase of the PDA shell thickness from a few nm to a maximum of about 50 nm [65], but also in an increase of the coating’s surface roughness. However, using dopamine concentrations lower than 0.5 g L^−1^ for functionalizing iron oxide nanoparticles allows the reduction of the formation of insoluble PDA aggregates formed during the synthesis, and the increase of PDA shell roughness. Another factor affecting the polymerization process is the type of buffer used. Recent studies have demonstrated that using Tris-HCl buffer instead of sodium bicarbonate (NaHCO_3_) and phosphate buffers, leads to the incorporation of Tris into the dopamine structure via covalent coupling between the primary amine of Tris and the dopamine-quinone intermediate, which is significant during the polymerization process [50]. The use of sodium hydroxide (NaOH) aqueous solutions instead of the above-mentioned buffers allows the preparation of PDA nanoparticles with good colloidal stability and a size lower than 100 nm. However, in all cases, the formation of large PDA aggregates can be observed, and additional purification steps are necessary for their removal from the final preparation [50]. In addition to these two parameters, the effect of the solution pH value must be considered. In fact, at basic pH, a consumption of the produced hydrogen protons can be observed as the PDA synthesis progresses, thus allowing the shifting of the redox equilibrium towards PDA production [50]. Therefore, an increase of the initial pH results in an increase of the PDA shell thickness in the case of coatings, and in a particle size reduction in the case of PDA nanoparticles production [50].

Owing to its described properties, polydopamine is an ideal candidate for the fabrication of hybrid materials with specific functionalities. The following sections will give some illustrative examples in order to describe the potential applications of polydopamine-coated magnetic iron oxide nanoparticles across the fields of bioremediation, biomedicine, and sensing.

## 4. Technological Applications of Polydopamine Functionalized Iron Oxide Nanoparticles

**Figure 2:**
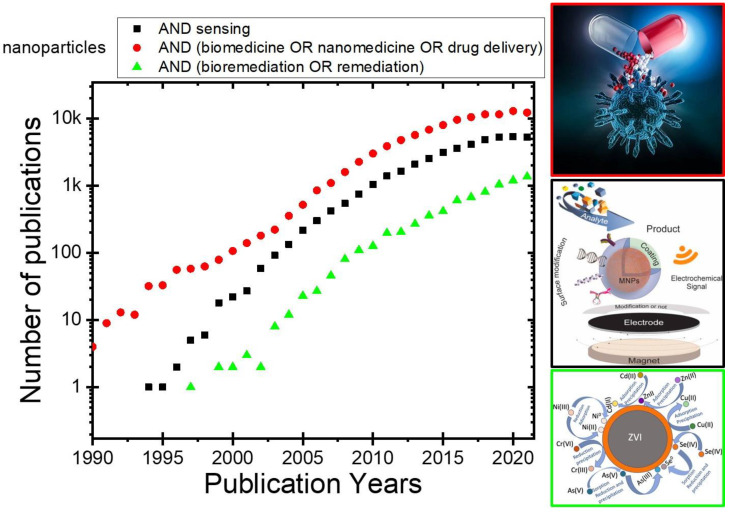
Number of publications with keywords: nanoparticles in combination with ■ sensing, ● biomedicine or nanomedicine or drug delivery, and ▲ bioremediation or remediation in the last years.

The use of nanoparticles for applications in the field of remediation, biomedicine, and sensing has been extensively researched in the last years, yielding significant advancement in the development of ultrasensitive nanosystems, as confirmed by the great number of publications (Figure 2). In this section, we will explore in particular recent advances in the applications of polydopamine-coated iron oxide nanoparticles.

### 4.1. Environmental Remediation

The removal of pollutants such as heavy metals, aromatic compounds, dyes, pesticides, and pharmaceuticals from water is a present challenge. Many methods have been exploited for the purification of water from contaminants, including photocatalytic degradation, biological treatments, adsorption, and chemical precipitation [66,67,68,69]. Among these, adsorption methods are still the most efficient thanks to their easy operation. In recent years, polydopamine-coated magnetic nanoparticles have emerged for the uptake of heavy metals in wastewater treatment, since they combine magnetic separation and a specific affinity towards pollutants. The functional groups of polydopamine offer a wide number of active sites for binding pollutants, and can selectively bind heavy metals via electrostatic interaction, hydrogen bonding, coordination, or chelation. Combining the chemical properties of polydopamine with the magnetic properties of magnetic nanoparticles is of great interest for adsorption applications, since magnetic materials can collect substances in water and be removed by the application of a magnetic field. Several works on the use of polydopamine-coated magnetic nanoparticles as adsorbent for pollutants have been reported.

**Figure 3:**
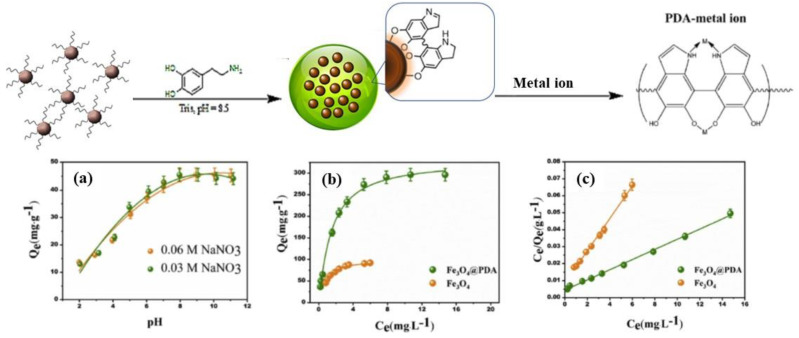
The structure of Fe_3_O_4_/PDA and its interaction with metal ions. Influence on adsorption of (**a**) pH and ionic strength; (**b**) concentration; (**c**) Langmuir model for Cd(II) adsorption, where Q_e_ (mg/g) is the equilibrium adsorption capacity and C_e_ is the Cd(II) concentration at the equilibrium conditions. Reproduced with permission from ref. [74]. Copyright 2019 Elsevier.

Among the toxic heavy metal ions, Cd(II) is considered to be an extremely harmful pollutant, and its accumulation in human body leads to many cardiovascular and neurological diseases [70]. Several methods have been employed for the removal of Cd(II) from polluted water [71,72,73]. In 2019, Sun et al. [74] proposed the synthesis of Fe_3_O_4_@PDA microspheres with strong saturation magnetism as adsorbents for Cd(II) from aqueous solution, displaying the high efficiency of the obtained material for the removal of the metal ions in a systematic study as a function of time, pH and ionic strength and concentration (Figure 3). They demonstrated the excellent performance of the nanosystem for Cd(II), thanks to its porous structure that allows the diffusion of Cd(II) and the subsequent removal of ions. Another example is the use of magnetic core-dual shell Fe_3_O_4_@PDA@TiO_2_ nanoparticles as adsorbent of U(VI) from aqueous solution under pH 3.0 and 8.2 as reported by Zhang et al. [75]. Desorption and reusability studies conducted on the developed nanoparticles confirmed the performance of Fe_3_O_4_@PDA@TiO_2_ as an efficient uranium adsorbent.

In addition to heavy metals, other pollutants have been considered for water remediation. For example, alginate beads with dispersed polydopamine CoFe_2_O_4_ particles were used for the removal of dyes such as methylene blue (MB), malachite green (MG), and crystal violet (CV) [76], exhibiting high adsorption performances due to the porous structure and large surface area. In particular, they found that the removal efficiency is higher in a pH range of 4.0–9.0 and a higher adsorption capacity towards MB and CV. Tan et al. [77] proposed the synthesis of heterostructure based on PDA-coated graphene oxide/Fe_3_O_4_ imprinted nanoparticles for selective adsorption of fluoroquinolone antibiotics by specific recognition and magnetic separation. The system had a large adsorption capacity (70.9 mg/g), deriving from the electrostatic interactions and the molecular recognition between the molecules and the PDA film, and it could be repeatedly used without loss of removal efficiency, which was higher than 95%.

The same system was used by He et al. [78] for the selective removal of sulfonylurea in cereals, leading to the possibility of using these nanoparticles for the selective detection and adsorption of herbicides in cereal samples by magnetic solid phase extraction coupled with High Performance Liquid Chromatography (HPLC). However, further investigation is required to improve the adsorption capacity of the proposed nanoparticles towards sulfonylurea. The above-mentioned applications of polydopamine-coated magnetic nanoparticles in the field of environmental remediation are reported in Table 1, and are highlighted for their great potential for the efficient removal and collection of heavy metals, antibiotics, dyes, and other organic pollutants present in environmental water.

### 4.2. Biomedicine

The superparamagnetic properties of iron oxide nanoparticles are also of great interest for applications in the biomedical field, including in therapeutic agents in cancer treatment (hyperthermia), drug delivery, biosensors, magnetic resonance imaging contrast agents, and cell separation, because they exhibit tuneable, size-dependent magnetic properties, and are benign and biodegradable [79,80,81]. On the other hand, polydopamine has been widely investigated for biomedical applications thanks to its biocompatibility and hydrophilicity. Moreover, its chemical groups allow subsequent chemical modification with functional groups for decoration with specific biomolecules, thus achieving original multifunctional hybrid nanosystems which are useful for biomolecular therapeutics and biomedicine [82]. Thus, the growth of a PDA shell on the nanocrystals’ (NCs) surface represents an efficient method for prevent NCs’ toxicity, to impart them with biocompatibility, and to introduce new functionalities on their surface useful for subsequent reactions.

Fe_3_O_4_@PDA nanoparticles have found several applications in the biomedical field, including in cancer diagnosis, photothermal therapy, bioimaging, and drug delivery. Photothermal therapy (PTT) is considered an alternative treatment to current cancer therapies such as radiotherapy, chemotherapy, and surgery, because it is highly selective and minimally invasive. It uses photosensitizing agents for absorbing the near-infrared (NIR) light, which is subsequently transduced into heat to destroy cancer cells [83].

**Figure 4:**
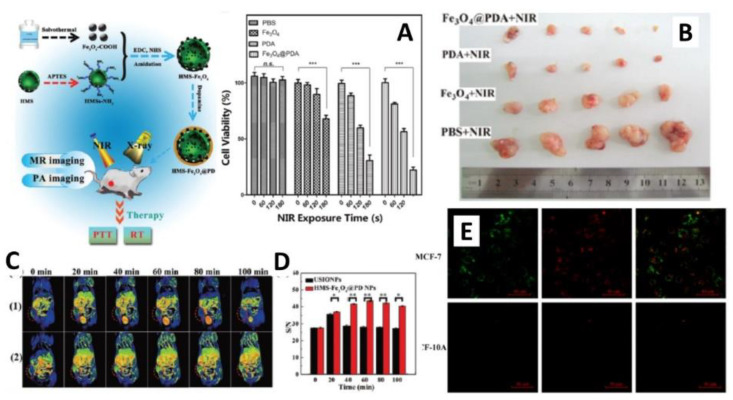
(**A**) Relative viabilities of A549 cells treated with Fe_3_O_4_, PDA, and Fe_3_O_4_@PDA at a concentration of 50 μg mL^−1^ without or with NIR laser irradiation; (**B**) Photograph of tumors after excision from PBS, Fe_3_O_4_, PDA, and Fe_3_O_4_@PDA under NIR irradiation (reproduced with permission from ref. [88]. Copyright 2015 ACS Publications); (**C**) T_1_-Weighted MR imaging of xenograft 4T_1_ tumors in mice before and at different time points post intravenous injection of ultrasmall iron oxide nanoparticles (USIONPs); (**D**) MR signal/noise (S/N) ratios of tumors at different time points (reproduced with permission from ref. [28]. Copyright 2013 Royal Society of Chemistry); (**E**) Simultaneous detection of multiple mRNAs in living cells (reproduced with permission from ref. [39]. Copyright 2014 ACS Publications).

Several studies have demonstrated that Fe_3_O_4_ nanoparticles can ablate tumors via photothermal effect thanks to their broad absorption in the NIR region and good light-thermal conversion efficiency [84]. Moreover, their superparamagnetic properties make Fe_3_O_4_ nanoparticles potentially useful as a magnetic resonance (MR) imaging contrast agent [85]. However, Fe_3_O_4_ nanoparticles need to be properly functionalized for applications in photothermal therapy against cancer, since they require a high iron oxide concentration to be utilized for PTT, exhibit a lack of efficient drug loading capability, and do not respond to stimuli such as pH and/or temperature for drug release [84,86]. The modification of magnetic nanoparticles with PDA was found to improve the efficiency of photothermal conversion and the NIR absorption, due to the strong near-infrared (NIR) light absorption property of PDA and its excellent photothermal conversion efficiency [87]. Moreover, the functional groups on the PDA surface can be exploited for the drug loading to perform effective chemotherapy. For instance, it was shown that increasing the thickness of PDA shell results in an increase in the efficacy of photothermal conversion and NIR absorption of Fe_3_O_4_@PDA particles [88] (Figure 4A,B). Several works have demonstrated that nanocomposites based on Fe_3_O_4_@PDA can be used as a cancer theranostic agent directed by a magnetic field, combining the highly sensitive MR capability of the superparamagnetic iron oxide nanoparticles (SPIONs) core and the cancer cell killing capability of the polydopamine coating by photothermal conversion effect [89]. However, the use of NPs as theranostic agents is limited because of their loss of stability in the blood circulation. Therefore, they can be superficially modified with polymeric moieties in order to improve their in vivo performance. In this perspective, a core-shell magnetite nanoclusters@PDA-PEG@ICG nanobead was synthetized and then conjugated with polyethylene glycol (PEG) in order to enhance the stability of NPs in the blood circulation [90]. The nanocomposites exhibited high accumulation in target tumors under the application of an external magnetic field, biocompatibility, and high T_2_ relaxivity in MRI imaging. The subsequent functionalization with indocyanine green (ICG) revealed a higher efficacy of photothermal conversion and a further photothermal effect in killing liver cancer cells under the irradiation of NIR laser. In another work, Shi et al. [28] fabricated a novel system based on polydopamine-coated magnetic mesoporous silica nanoparticles for multimode cancer theranostics. In particular, they used SPIONs for T_1_ weighted MR imaging, and PDA shell was used to impart a good colloidal stability and to absorb NIR light for the photothermal therapy of tumors (Figure 4C,D). The prepared nanoparticles exhibited NIR absorption, enabling their possible use for multifunctional T_1_ MR, thermal imaging, and PTT of xenografted tumor models. In 2020, Jędrzak et al. [91] designed a multimodal nanoplatform based on polydopamine (PDA)-coated magnetite nanoparticles (NPs) and spheres (sMAG) with PAMAM dendrimers for the treatment of hepatocellular carcinoma (HCC) in vitro. They proved that these nanoplatoforms, functionalized with NHS-PEG-Mal (*N*-hydroxysuccinimide–polyethylene glycol–maleimide) linker terminated with folic acid, can be used as efficient agents for dual chemo and photothermal therapy of HCC. Very recently, Jin et al. [92] developed a multifunctional porous Fe_3_O_4_@PDA-PEG nanocomposite that simultaneously can serve for magnetic resonance (MR) imaging, photothermal therapy (PTT), and chemotherapy, and can potentially be used as a theranostic agent for biomedical applications. Thanks to the porous structure of iron oxide nanoparticles and the functional groups on the surface of PDA, a remarkable drug loading capacity was observed, while the photothermal-chemotherapy showed an enhanced anti-tumor effect during in vitro experiments.

Magnetic nanomaterials and the unique properties of PDA are also of great interest for the development of drug delivery systems. Thus, researchers are recently working on the functionalization of these nanoplatforms with different types of drug molecules. Liu et al. [93] proposed the synthesis of core-shell Fe_3_O_4_ polydopamine NPs as a drug carrier, exploiting the catechol groups on PDA surface to create a pH responsive drug carrier system. Fe_3_O_4_@PDA nanoparticles were loaded with the anticancer drug bortezomib (BTZ) in order to investigate the pH-responsive drug release behavior of NPs, and they were demonstrated to be effective at controlling the release of BTZ in a pH-sensitive manner. In another work, Fe_3_O_4_@PDA core-shell nanocomposites were used as theranostics agents for intracellular mRNA detection. They showed that PDA can adsorb dye-labelled single-stranded DNA (ssDNA) probes and quench the fluorescence of the dye. In the presence of the target, the binding between the dye-labelled ssDNA probe and its target form a duplex structure, leading to a release of the probe from PDA and recovery of the fluorescence. In this way, Fe_3_O_4_@PDA NCs could be used as a nanoprobe to detect mRNA in living cells (Figure 4E) [39]. Singh et al. [94] proposed the development of nanocarriers based on PDA-coated iron oxide nanoparticles (IONPs) functionalized with Glutathione disulfide (GSSG), which acts as a cellular trigger to release the drug from the nanoparticles, for the treatment of prostate cancer. Release studies performed on Doxorubicin (DOX), an anticancer drug widely used in cancer treatment, showed a pH-responsive behavior and a decrease of several side effects, making the elaborated nanocarrier a potential drug delivery system.

In 2020, Singh designed PDA-coated iron oxide nanorods conjugated with taurine—a biomolecule that can improve the performance of nanodelivery vehicles as it can cross the blood-brain barrier (BBB)—as an agent for cancer therapy. DOX was loaded on the nanovehicles, showing also in this case that the behavior of the nanocarrier depends on the pH value, making them an efficient tool for effective delivery in the tumor microenvironment. In vitro studies performed on prostate carcinoma cells (PC3) revealed the good cellular uptake of the nanohybrid which can induce the cell death and potentially be used as a nanocarrier for the treatment of cancer cells [95]. All mentioned studies are summarized in Table 2.

### 4.3. Sensing

Miniaturized (bio)sensors have been widely investigated in the past years as a way to achieve real-time monitoring and implement automated lab on chip platforms, exploiting their high selectivity and sensitivity and integration in portable measurements systems [96,97,98,99]. An important component for the operation of a biosensor is the immobilization of biorecognition probes, since it affects the sensitivity, the selectivity, and the stability of biosensors.

In this perspective, polydopamine was demonstrated to be a useful biopolymer to immobilize many biomolecules on electrodes while preserving their biological activity. By changing the immobilized molecules, several high performance polydopamine-containing biosensors for various electrochemical assays have been engineered. For example, in 2013, a biosensor based on multi-functional core-hell glucose oxidase–Au–PDA–Fe_3_O_4_ magnetic nanoparticles has been proposed by Peng et al. [100] for glucose detection. They demonstrated that the modified electrodes preserved the native structure of the immobilized proteins, and had a good electrocatalytic activity for the oxidation of glucose. Moreover, the entrapped glucose oxidase preserves its bioactivity thanks to the high biocompatibility of PDA and the ability of the system in efficiently communication with electrodes.

**Figure 5:**
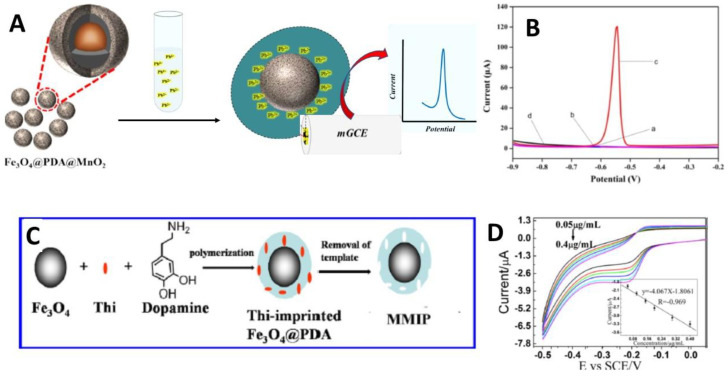
(**A**) Capture and detection of the target Pb(II) in sample solution; (**B**) Differential pulse voltammograms of the developed nanocomposites (reproduced with permission from ref. [101]); (**C**) Schematic illustration of the fabrication process of Fe_3_O_4_NPs MMIPs; (**D**) Cyclic voltammetry from Fe_3_O_4_NPs MMIPs-modified glassy carbon electrode (GCE) dose dependent (reproduced with permission from ref. [102]. Copyright 2017 Elsevier).

Polydopamine-based electrochemical sensors for metal ions are also common. Wang and co-workers explored the absorbent properties of Fe_3_O_4_@polydopamine-MoS_2_ core-shell nanospheres for sensitive electrochemical detection of Pb^2+^ in environmental samples [101]. They found a fast adsorption and high adsorption capacity of nanospheres to Pb^2+^, in addition to their easy separation from water by an external magnetic field. Polydopamine coating has been used both for protecting the magnetic core and as a template for the in-situ growth of MoS_2._ The adsorption capacity of nanospheres was observed to be dependent on solution pH. In particular, increasing the solution pH in range of 1.0–4.0 results in a great adsorption of Pb^2+^ on the surface of Fe_3_O_4_@PDA-MoS_2_ nanospheres due to increased electrostatic interactions between the nanocomposite surface and lead ions. (Figure 5A,B).

Recently, many researchers have focused their work on detection techniques based on molecular imprinting polymers (MIPs) as artificial receptors for a target molecule based on synthetic polymers. The preparation procedure involves the presence of a template molecule around which interacting and cross-linking monomers are arranged and co-polymerized to form a cast-like shell. Specifically, cavities are imprinted in the polymer, which can now selectively recognize the target through steric and binding interactions. MIPs thus represent an alternative to natural receptors, with the main advantages being robustness, versatility, and cost effectiveness. For these reasons MIPs are receiving remarkable attention as smart and robust materials for applications such as affinity separation [103], chemical sensors and assay [104], solid-phase extraction [105], catalysis [106], artificial enzyme inhibitor/antibody [107], and drug delivery [108]. In this perspective, polydopamine is very attractive as a promising molecular imprinted polymer for biosensing thanks to its excellent biocompatibility, hydrophilicity, chemical functionalities, robust adhesion to various substrates, and controllable thickness [109,110].

Liu et al. [111] first reported the use of a polydopamine imprinted film for the capacitive detection of biomolecules. Zhou et al. [112] reported the deposition of a thin PDA shell on Fe_3_O_4_ nanoparticles using human hemoglobin as template molecule. Five different non-template proteins were used to test capacity of the imprinted Fe_3_O_4_@polydopamine nanoparticles to recognize the target protein, showing a high recognition capacity of haemoglobin imprinted Fe_3_O_4_@polydopamine NPs towards haemoglobin. The obtained results indicated that the imprinted Fe_3_O_4_@polydopamine NPs, in conjugation with strong magnetism, could be used as affinity materials for the selective recognition and separation of target proteins. In 2017, Wang et al. [102] prepared for the first time a new nanoenzyme of Fe_3_O_4_ nanoparticles (NPs) magnetic molecularly imprinted polymers (MMIPs), by polymerizing dopamine on the Fe_3_O_4_NPs surface in the presence of thionine (Thi) as template (Figure 5C,D). The results showed that the imprinting sites improved the selectivity of Fe_3_O_4_ NPs MMIPs greatly. Moreover, they found that Fe_3_O_4_NPs MMIPs could selectively catalyze the reduction of Thi in the presence of H_2_O_2_. Accordingly, they proposed Fe_3_O_4_ NPs MMIPs by using Thi as probe for the fabrication of a highly selective and sensitive electrochemical H_2_O_2_ biosensor that could be detect acetylthiocholinechloride (AChl), acetylcholinesterase (AChE), and the choline oxidase (ChOx).

Very recently, Miao et al. [113] designed a highly selective impedance chemical sensor based on Fe_3_O_4_ and PDA molecularly imprinted polymer magnetic nanoparticles (PDA@Fe_3_O_4_ MIP MNPs) for the ultrasensitive detection of dichlorodiphenyltrichloroethane (DDT) in food samples. They showed that PDA@Fe_3_O_4_ MIP MNPs could specifically recognize and efficiently adsorb and extract 4,40-DDT from food samples. Moreover, the application of an external magnetic field easily allows the separation of DDT. After the adsorption of DDT, the electrochemical impedance value of the PDA@Fe_3_O_4_-MIP MNPs increased sensitively, indicating the correlation between the impedance response and the amount of analyte. Using this novel sensor, the selective recognition of a wide range of molecules could be achieved. Table 3 summarizes the above-mentioned applications of Fe_3_O_4_@PDA nanocomposites in the field of sensing.

## 5. Conclusions

This review illustrates that the magnetic properties of iron oxide nanoparticles and the singular properties of polydopamine are of great interest for applications in biomaterials science. Dopamine can easily modify iron oxide nanoparticles, allowing the best stability and the introduction of new functionalities. Thanks to their long-term stability and resistance to oxidation, such nanomaterials can be utilized in different areas, from environmental to biomedical applications. Future research should be directed towards the testing of these systems in more complex conditions (water samples with different contaminants, origin of samples, etc.) and in physiological environments. The application of Fe_3_O_4_@PDA nanosystems in biomedical areas should completely provide in vivo and in vitro assays in order to highlight their potential usefulness in cancer therapy. Thus, more research on the application of PDA-coated iron oxide nanoparticles and specific interdisciplinary studies on the modification and the functionality of this class of nanosystems are expected to further prove their potential.

## Figures and Tables

**Table 1:** Fe_3_O_4_@PDA nanocomposites for the uptake of organic and inorganic pollutants.

Biosorbent	Pollutants	Adsorption Capacity (mg/g)	pH	Reference
PDA-coated graphene oxide/Fe_3_O_4_ imprinted nanoparticles	Sulfonylurea	3.176	-	[78]
Sodium alginate@CoFe_2_O_4_-PDA beads	Malachite green	248.8		
	Crystal violet Methylene blue	456.5 466.6	4	[76]
PDA-coated graphene oxide/Fe_3_O_4_ imprinted nanoparticles	fluoroquinolone antibiotics	70.90	8	[77]
Fe_3_O_4_@PDA@TiO_2_ nanoparticles	U(VI)	87.74	8.2	[75]
Fe_3_O_4_@PDA microspheres	Cd(II)	296.4	6	[74]

**Table 2:** Fe_3_O_4_@PDA nanocomposites for biomedical applications.

Bionanocomposite	Application	Reference
Core-shell Fe_3_O_4_ polydopamine nanoparticles	pH responsive drug delivery	[93]
Core-shell Fe_3_O_4_ polydopamine nanoparticles	Intracellular mRNA detection	[39]
Nanoclusters@PDA-PEG@ICG	Cancer therapy	[90]
Polydopamine-coated magnetic mesoporous silica nanoparticles	Multimode cancer theranostic	[28]
IONPs@PDA	Drug delivery system for cancer therapy	[94]
Polydopamine (PDA)-coated magnetite nanoparticles (NPs) and spheres (sMAG) with PAMAM dendrimers	Hepatocellular carcinoma treatment	[91]
PDA-coated iron oxide nanorods	Drug delivery system for cancer therapy	[95]
Porous Fe_3_O_4_@PDA-PEG nanocomposite	Magnetic resonance (MR) imaging Photothermal therapy (PTT) Chemotherapy	[92]

**Table 3:** Fe_3_O_4_@PDA nanocomposites for sensing applications.

Bionanocomposite	Application	Analyte	Reference
Fe_3_O_4_@PDA nanoparticles	Recognition and separation	Haemoglobin	[112]
Core–shell glucose oxidase–Au–PDA–Fe_3_O_4_ nanoparticles	Glucose sensor	Glucose	[100]
PDA@Fe_3_O_4_ MIP (Molecularly Imprinted Polymer)	Electrochemical biosensor	Thionine	[102]
Fe_3_O_4_@PDA@MnO_2_	Electrochemical sensor	Pb^2+^	[101]
PDA@Fe_3_O_4_ MIP	Impedance sensor	Dichlorodiphenyltrichloroethane (DDT)	[113]

## Data Availability

Data sharing not applicable.
